# Global Postural Reeducation for patients with musculoskeletal conditions: a systematic review of randomized controlled trials

**DOI:** 10.1590/bjpt-rbf.2014.0153

**Published:** 2016-04-01

**Authors:** Giovanni E. Ferreira, Rodrigo G. P. Barreto, Caroline C. Robinson, Rodrigo D. M. Plentz, Marcelo F. Silva

**Affiliations:** 1 Programa de Pós-graduação em Ciências da Reabilitação, Universidade Federal de Ciências da Saúde de Porto Alegre (UFCSPA), Porto Alegre, RS, Brasil; 2 Programa de Pós-graduação em Fisioterapia, Universidade Federal de São Carlos (UFSCar), São Carlos, SP, Brasil; 3 Programa de Pós-graduação em Ciências da Saúde, Universidade Federal de Ciências da Saúde de Porto Alegre (UFCSPA), Porto Alegre, RS, Brasil

**Keywords:** global posture reeducation, systematic review, physical therapy, low back pain, neck pain, ankylosing spondylitis

## Abstract

**Objectives:**

To systematically review randomized controlled trials that assessed the effects of Global Postural Reeducation (GPR) on patient-reported outcomes in conditions of the musculoskeletal system.

**Method:**

An electronic search of MEDLINE (via PubMed), EMBASE, Cochrane CENTRAL, and SciELO was performed from their inception to June 2015. Randomized controlled trials that analyzed pain and patient-reported outcomes were included in this review. The Cochrane Collaboration’s Risk of Bias Tool was used to evaluate risk of bias, and the quality of evidence was rated following the GRADE approach. There were no language restrictions.

**Results:**

Eleven trials were included totaling 383 patients. Overall, the trials had high risk of bias. GPR was superior to no treatment but not to other forms of treatment for pain and disability. No placebo-controlled trials were found.

**Conclusion:**

GPR is not superior to other treatments; however, it is superior to no treatment. Due to the lack of studies, it is unknown if GPR is better than placebo. The quality of the available evidence ranges from low to very low, therefore future studies may change the effect estimates of GPR in musculoskeletal conditions.

## Bullet points

- To date, the effects of GPR on patient-reported outcomes have not been summarized.
- GPR is better than no treatment.
- GPR is not better than other treatments.
- The quality of the available evidence ranged from low to very low quality.
- Future trials must improve the reporting quality and reduce bias.

## Introduction

Disorders of the musculoskeletal system represent a high societal and economic burden, accounting for a high prevalence of disability. In disorders such as low back pain^1^, ankylosing spondylitis^2^ ^,^ ^3^, neck pain^4^, and temporomandibular disorders^2^ ^,^ ^5^ ^,^ ^6^, physical therapy has been recommended as a first-line treatment.

Among the various methods of treatment, Global Postural Reeducation (GPR) is of particular interest. This method was empirically developed by Phillippe Souchard in 1981^7^ and is currently used in countries like Brazil, Spain, France, and Portugal^8^ ^,^ ^9^. The philosophy of GPR lies in three fundamental principles: (1) Individuality, which considers each person as unique; (2) Causality, which states that the true cause of a musculoskeletal condition may arise from distant sites; and (3) Totality, which determines that a body should be treated in its entirety. Moreover, GPR considers the existence of different muscle chains (i.e. a series of interconnected muscles constituting a continuum along the body that play specific functional roles)^10^ ^,^ ^11^. The main muscle chains are the posterior static chain and the anterior diaphragmatic chain. Based on these principles, it is assumed that pathological conditions may arise due to retractions in the muscle chains. Thus, each patient is treated individually with specific static postures in order to stretch the shortened muscle chains and to enhance co-contraction of the antagonists. By stretching the shortened muscles and enhancing the contraction of the antagonists, the ultimate goal of this approach is to improve postural symmetry, which is believed to mediate the reduction of pain and disability.

The clinical effects of GPR have been investigated on conditions such as temporomandibular disorders, neck pain, ankylosing spondylitis, and low back pain. A literature review^8^ published in 2011 identified thirteen papers, among which only four were randomized controlled trials that addressed the influence of GPR on patient-reported outcomes. This review was inconclusive, as some studies showed positive results, while others did not. But the conclusions of this review were drawn based on the results of different study designs on patient-reported outcomes and surrogate outcomes without distinction. This approach hampered a correct judgement regarding the clinical effect of GPR, as surrogate outcomes are potentially misleading and may not predict clinically important outcomes accurately^12^.

In this sense, there is a need for a systematic review in order to provide an accurate perspective on the current evidence concerning the effects of GPR on conditions of the musculoskeletal system. The conduction of a systematic review including only randomized controlled trials is crucial, as this is the only study design that allows controlling of confounders, such as the natural history, regression to the mean, the Hawthorne effect, placebo effects, among others^13^. The goal of this paper is to systematically review randomized controlled trials that assessed the effects of GPR on conditions of the musculoskeletal system.

## Method

This systematic review followed the PRISMA recommendations^14^ ^,^ ^15^ as well as the tutorial for writing systematic reviews of the Brazilian Journal of Physical Therapy^16^. The study was prospectively registered under the identifier: CRD42014013787 (PROSPERO).

### Literature search strategy

MEDLINE via PubMed, EMBASE, Cochrane CENTRAL, and SciELO were systematically investigated from their inception to June, 2015. Grey literature was searched through OpenGrey and Scholar Google. Websites of the Brazilian and Spanish associations of GPR, as well as specific GPR websites from France and USA were screened. The reference list of the included trials were also screened. There were no restrictions regarding language during the search phase. The search strategy for PubMed is depicted in Appendix 1.

### Eligibility criteria

This review included randomized controlled trials that used GPR as a treatment method in individuals with age ≥18 years with any condition affecting the musculoskeletal system published in English, Portuguese, French, and Spanish. The included studies had to compare GPR to no intervention, any other intervention, or *sham* intervention and had to assess patient-reported outcomes, defined as reports coming directly from patients about how they feel or function in relation to a health condition and its therapy without interpretation by healthcare professionals or any other individual^12^, with any validated outcome measure.

### Study selection

Two independent reviewers (G.F. and R.B.) screened titles and abstracts. Differences were solved by consensus. In the absence of consensus, a third reviewer arbitrated (M.S.).

### Risk of bias

Two reviewers (G.F. and R.B.) independently rated risk of bias with the Cochrane Collaboration’s Tool for assessing risk of bias^17^. This instrument has six domains: selection bias (random sequence generation and allocation concealment), performance bias (blinding of participants and personnel), detection bias (blinding of outcome assessment), attrition bias (incomplete data outcome), reporting bias (selective reporting), and other biases. Each item was rated as low, high, or unclear risk of bias.

### Data extraction and analysis

Data were independently extracted by two reviewers (G.F. and R.B.) to a spreadsheet containing characteristics of the individuals enrolled, interventions, and comparators. A third reviewer (C.R.) checked the data and arbitrated any disagreements.

### Quality of evidence

The quality of evidence across studies followed the principles of the GRADE approach^18^. The GRADE comprises five items: (1) presence of within-study limitations (risk of bias); (2) inconsistency of results (heterogeneity); (3) indirectness of evidence; (4) imprecision of the effect estimates; (5) risk of publication bias. Each non-satisfied item downgraded the overall quality of evidence for each outcome. The quality of the evidence was classified into four categories: high, moderate, low, and very low. When an outcome was assessed by only one study, the overall quality was initially considered low and the presence of high risk of bias downgraded the quality of evidence to very low^19^.

### Data analysis

All outcomes reported were considered to be primary. For each outcome, point estimates and its respective 95% confidence intervals were calculated in Review Manager Version 5.2 (The Nordic Cochrane Centre, The Cochrane Collaboration). As the calculated estimates were based on raw means and standard deviations, slight differences were noted in some publications^4^ ^,^ ^20^ ^-^ ^22^. A descriptive analysis was employed, since pooling revealed high levels of statistical heterogeneity (I^2^ >90% regardless of the chosen effect measure).

## Results

### Study Selection

**Figure 1:**
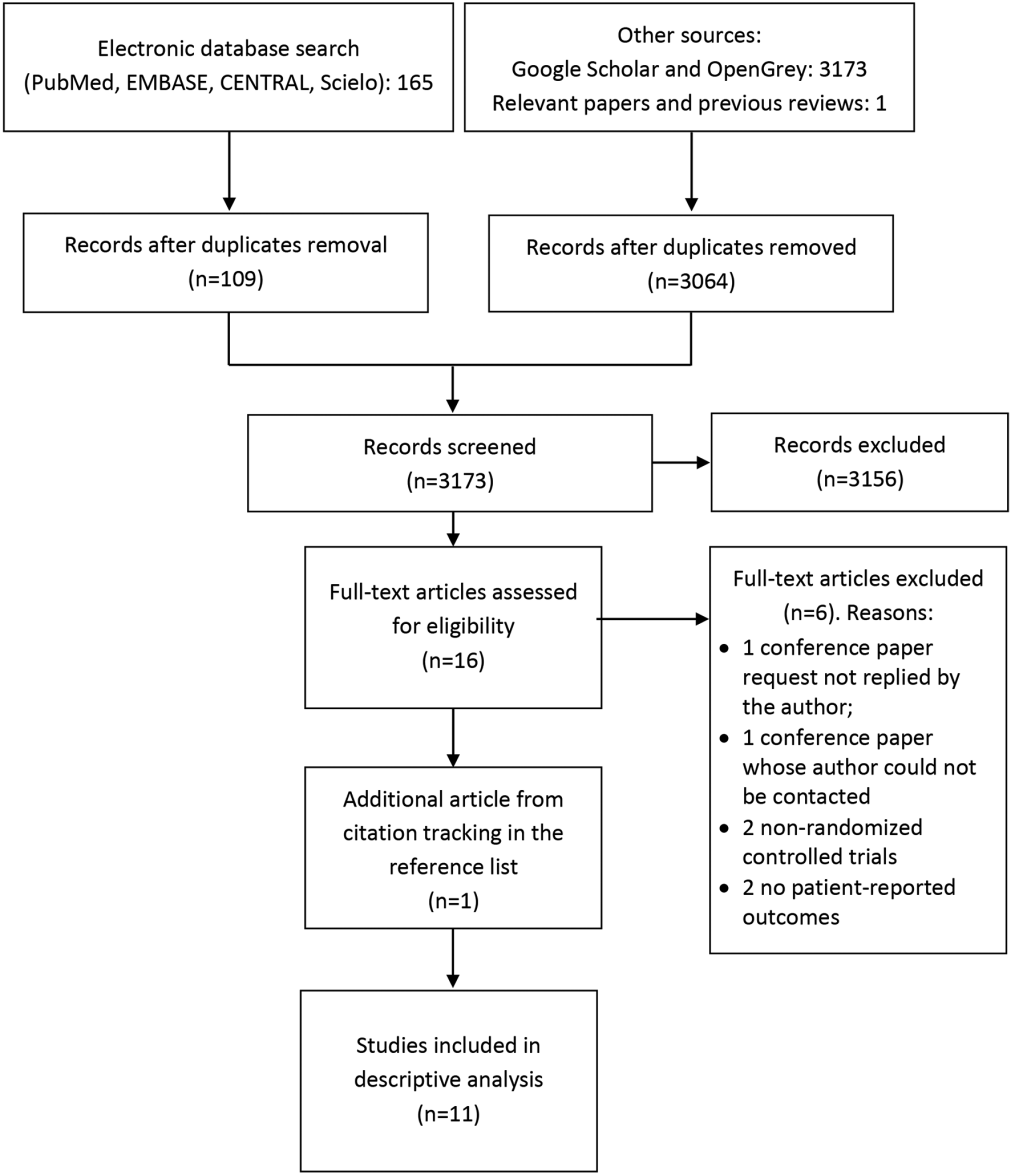
PRISMA flow diagram.

Up to June 2015, the database search retrieved 165 articles. After duplicates removal, 109 titles and abstracts were screened for eligibility. Of those, 16 were selected for full-text reading. Two conference abstracts were screened^23^ ^,^ ^24^ but eventually excluded since the authors could not be contacted. After four additional exclusions^25^ ^-^ ^28^, 10 trials were included in this step. Grey literature yielded 3173 citations, none of which were additional randomized controlled trials. We therefore concluded that the probability of publication bias was reduced. There were no exclusions due to language restrictions^29^. Citation tracking in the reference list of potential papers and a previous review^8^ found one additional trial^20^. Thus, 11 trials were included in this systematic review. The study flow diagram is depicted in Figure 1.

### Study characteristics

Table 1 outlines the characteristics of the included studies. This review included 383 participants (mean of 35 patients per trial, ranging from 26 to 61 patients). Of these, 355 participants presented follow-up data (92.68%). A mean of 10 weeks of treatment were provided among studies, ranging from six^4^ to 16 weeks^30^. A mean of 15 treatment sessions were provided, ranging from eight^5^ ^,^ ^22^ to 60^21^. In ten trials^4^ ^,^ ^5^ ^,^ ^20^ ^,^ ^22^ ^,^ ^30^ ^-^ ^35^, GPR was supervised by a physical therapist. Only Durmus et al.^21^ engaged patients in a home exercise program without supervision. Eight trials^5^ ^,^ ^22^ ^,^ ^30^ ^-^ ^35^ delivered treatment sessions once a week, while two trials^4^ ^,^ ^20^ treated patients twice a week.

**Table 1:** Study characteristics.

**Author (condition)**	**N (follow-up)**	**IG/CG**	**Intervention description**	**Intervention duration (weeks)**	**Total sessions**	**Comparison group**	**Outcome measures**	**Follow-up time**
Adorno and Brasil-Neto^31^ (chronic back pain)	30 (30)	10/10/10	Global postural reeducation (GPR) for anterior internal muscle strings of the hip, the posterior master string and the respiratory string.	12	12 (once a week)	Isostretching: pelvis repositioning, lowering of the scapula (isometric fixation) and self-growth of the spine	VAS	12 and 20 weeks
Amorim et al.^32^ (chronic neck pain)	36 (30)	18/18	Global stretching for anterior and posterior chains	10	10 (once a week)	Segmental stretching (passive stretching exercises for the head, cervical spine and upper limbs)	DASH/NDI/ VAS	10 weeks
Cabral et al.^20^ (patellofemoral pain syndrome)	26 (20)	10/10	Global stretching for the posterior chain	8	16 (twice a week)	Segmental stretching (hamstrings, gastrocnemius)	VAS, LKQ	8 weeks
Cunha et al.^4^ (neck pain)	31 (31)	15/16	2 postures in supine position with stretching for posterior and anterior muscle chain	6	12 (twice a week)	Segmental stretching (upper trapezius, sub occipital, pectoralis major and minor, rhomboids, finger and wrist flexors, forearm pronators, finger and wrist extensors, forearm supinators, and paravertebral muscles)	VAS	6 weeks and 12 weeks
Durmus et al.^21^ (ankylosing spondylitis)	56 (51)	20/21/15	GPR + mobility exercises + breathing exercises	12	60	Mobility exercises, motor control exercises, segmental stretching, neural mobilization, abdominal strengthening and breathing exercises; No treatment	VAS/BASFI/ BASDAI/	12 weeks
Fernandez-de-las-Peñas et al.^33^ ^,^ ^34^ (ankylosing spondylitis)a,b*	43 (40)	20/20	Global stretching for anterior and posterior chains	15	15 (once a week)	Mobility exercises, motor control exercises, segmental stretching, neural mobilization, abdominal strengthening and breathing exercises	BASMI/BASFI/BASDAI	16 weeks and one year
Gil et al.^22^ (pregnancy-related back pain)	34 (34)	17/17	Global stretching for posterior chain and internal-anterior chain of the upper limb	8	8 (once a week)	No treatment	VAS/RMQ	8 weeks
Lawand et al.^35^ (chronic low back pain)	61 (60)	31/30	GPR using all of the six postures described by Souchard.	12	12 (once a week)	No treatment	VAS/RMQ/BDI	12 weeks and 6 months
Maluf et al.^5^ (temporomandibular dirosders)	28 (24)	14/14	Global stretching for anterior and posterior chains	8	8 (once a Week)	Segmental stretching	VAS (TMJ, neck and headache)	8 weeks and 16 weeks
Silva et al.^30^ (ankylosing spondylitis)	38 (35)	22/16	Tailored to individuals shortened muscle chains	16	16 (once a week)	Segmental stretching + breathing exercises	VAS/BASDAI/ HAQ-S	16 weeks

N: sample size; IG: intervention group; CG: control group; GPR: Global Posture Reeducation; VAS: visual analogue scale; DASH: Disability of the Arm, Shoulder and Hand; NDI: Neck Disability Index; LKQ: Lysholm Knee Questionnaire; BASFI: Bath Ankylosing Spondylitis Functional Index; BASDAI: Bath Ankylosing Spondylitis Disease Activity Index; BASMI: Bath Ankylosing Spondylitis Metrology Index; RMQ: Roland-Morris Questionnaire; BDI: Beck Depression Inventory; TMJ: temporomandibular joint; HAQ-S: Health Assessment Questionnaire - Spondyloarthropaties.

* Two studies conducted in the same population, with the same procedures and outcomes, but analyzing different follow-ups.

### Risk of bias

Eight studies reported adequate randomization procedures^4^ ^,^ ^5^ ^,^ ^22^ ^,^ ^31^ ^-^ ^35^, whereas only three studies^5^ ^,^ ^32^ ^,^ ^35^ reported adequate allocation concealment procedures. Four studies^22^ ^,^ ^33^ ^-^ ^35^ adequately reported blinding of the outcome assessor. Seven studies^4^ ^,^ ^5^ ^,^ ^21^ ^,^ ^30^ ^,^ ^32^ ^-^ ^34^ had sample loss but did not perform intention-to-treat analysis and were therefore classified as having high risk of bias for incomplete outcome data reporting. Only one trial performed intention-to-treat analysis^35^. Only one trial was prospectively registered^35^. Ten studies were considered to have high risk of bias due to the lack of at least one of the following items: allocation concealment, blinding of the outcome assessor, or intention-to-treat analysis, and only one trial was considered to have low risk of bias^35^ (Table 2).

**Table 2:** Risk of bias summary. “Low” denotes low risk of bias; “High” denotes high risk of bias; “Unclear” denotes unclear risk of bias.

**Author**	**Bias domains**					
	Randomization	Allocation concealment	Blinding (participants and personnel)	Blinding (outcome assessment)	Incomplete outcome data	Selective reporting
Adorno and Brasil-Neto^31^	**Low**	**Low**	**High**	**Low**	**Low**	**Low**
Amorim et al.^32^	**Low**	**Low**	**High**	**Unclear**	**High**	**Low**
Cabral et al.^20^	**Unclear**	**High**	**High**	**Unclear**	**Unclear**	**Unclear**
Cunha et al.^4^	**Low**	**High**	**High**	**High**	**High**	**Low**
Durmus et al.^21^	**Unclear**	**High**	**High**	**High**	**High**	**Low**
Fernandez-de-las-Peñas^33^	**Low**	**High**	**High**	**High**	**High**	**Low**
Fernandez-de-las-Peñas et al.^34^	**Low**	**High**	**High**	**High**	**High**	**Low**
Gil et al.^22^	**Low**	**High**	**High**	**Low**	**Low**	**Low**
Lawand et al.^35^	**Low**	**Low**	**High**	**Low**	**Low**	**Low**
Maluf et al.^5^	**Low**	**Low**	**High**	**High**	**High**	**Low**
Silva et al.^30^	**Unclear**	**High**	**High**	**High**	**High**	**Low**

### GPR versus no treatment

Three studies comprising 136 individuals compared GPR versus no treatment for pregnancy-related low back pain^22^, chronic low back pain^35^, and ankylosing spondylitis^21^ (Table 3). Of these, 126 individuals presented follow-up data (92.64%). Two trials had high risk of bias and one had low risk of bias^35^.

**Table 3:** Results and conclusions of studies of GPR versus (A) no treatment; (B) segmental stretching; (C) other treatments.

**Author (condition)**		**Time point**		**Results**		**Conclusions**	
**A. GPR versus no treatment**							
Durmus et al.^21^ (ankylosing Spondylitis)		12 weeks		Pain (0-10): MD –2.90 (95% CI –3.99 to –1.80) favoring GPR^*^		GPR significantly reduced pain and disease activity and improved functional capacity compared to no intervention.	
				Functional capacity (0-100)^1^: MD 1.02 (95% CI –2.15 to 0.11) favoring GPR			
				Disease activity^2^ (0-10): MD –0.98 (95% CI -1.57 to –0.38) favoring GPR^*^			
Gil et al.^22^ (pregnancy-related back pain)		8 weeks		Pain (0-10): MD –5.5 (95% CI –6.08 to –4.91) favoring GPR^*^		GPR significantly reduced pain and disability compared to no intervention.	
				Disability^3^: MD -9.10 (95% CI –11.09 to –7.10) favoring GPR^*^			
Lawand et al.^35^ (chronic low back pain)		12 weeks		Pain (0-10): MD –3.1 (95% CI –3.79 to –2.40) favoring GPR*		GPR significantly reduced pain and disability and improved some domains of SF-36 (vitality, emotional aspects and mental health) compared to no intervention.	
				Disability (0-24): MD –4.4 (95% CI –6.05 to –2.74) favoring GPR^*^			
		6 months		Pain (0-10): MD –1.5 (95% CI –2.16 to –0.83) favoring GPR^*^			
				Disability (0-24): MD –4 (95% CI –5.8 to –2.18) favoring GPR^*^			
**B. GPR versus segmental stretching**							
Amorim et al.^32^ (neck pain)		10 weeks		*Pain (0-10):* MD –2.06 (95% CI –3.05 to –1.06) favoring GPR^*^		GPR reduced pain and disability compared to segmental stretching alone.	
				*Disability (0-50)* ^1^: MD –7.3 (95% CI –12.16 to –2.61) favoring GPR^*^			
Cunha et al.^4^ (neck pain)		6 weeks		*Pain (0-10)*: MD 1.0 (95% CI 0.04 to 1.96) favoring SS^*^		Segmental stretching alone was significantly better than GPR for pain reduction immediately and after six weeks.	
		12 weeks		*Pain (0-10)*: MD 1.1 (95% CI 0.05 to 2.14) favoring SS^*^			
Cabral et al.^20^ (patellofemoral pain)		8 weeks		*Pain (0-10)*: MD 0.7 (95% CI –2.64 to 0.12) favoring GPR;		GPR did not significantly reduce pain or disability compared to segmental stretching.	
				*Disability (0-100)* ^2^: MD –5.5 (95% CI –14.63 to 3.63) favoring the control condition			
Maluf et al.^5^ (temporomandibular disorder)		8 weeks		*Pain (0-10)*		GPR did not reduce TMJ pain compared to segmental stretching. Conversely, segmental stretching was more effective than GPR in reducing headache intensity at 8 weeks.	
				TMJ: MD –0.5 (95% CI –1.68 to 0.62) favoring GPR			
				Neck pain: MD –0.4 (95% CI –1.79 to 0.85) favoring GPR			
				Headache: MD 1.50 (95% CI 0.24 to 2.75) favoring SS^*^			
		16 weeks		*Pain (0-10)*			
				TMJ: MD –0.98 (95% CI –2.11 to 0.15) favoring GPR			
				Neck pain: –1.25 (95% CI –2.52 to 0.02) favoring GPR			
				Headache: MD –0.23 (95% CI –1.43 to 0.97) favoring GPR			
**C. GPR versus other treatments**						
Adorno et al.^31^ (back pain)	12 weeks		Pain (0-10): MD 0.7 (95% CI –2.44 to 1.04) favoring Isostretching;		GPR did not significantly reduce pain immediately and at three months follow-up compared to Isostreching.	
	20 weeks		Pain (0-10): MD –1.2 (95% CI –2.8 to 0.43) favoring GPR;			
Durmus et al.^21^ (Ankylosing Spondylitis)	12 weeks		Pain (0-10):MD –0.46 (95% CI –1.48 to 0.56) favoring GPR;		GPR did not significantly reduce pain and functional capacity compared to conventional exercise regimen, but significantly reduced disease activity.	
			Functional capacity^1^ (0-10): MD –0.01 (95% CI –0.65 to 0.62) favoring GPR			
			Disease activity^2^ (0-10): MD –3.63 (95% CI –4.71 to –2.56) favoring GPR^*^			
Fernandez-de-las-Peñas et al.^33^ (Ankylosing Spondylitis)	16 weeks		*Functional capacity* *^3^* (0-10): MD –0.42 (–1.05 to 0.20) favoring GPR *Disease activity* *^2^* (0-10): MD 0.12 (95% CI –0.50, 0.74) favoring conventional physical therapy		GPR did not significantly reduce disease activity nor improve pain compared to conventional exercise regimen, immediately and at one year follow-up.	
		
Fernandez-de-las-Peñas et al.^34^ (b) (Ankylosing Spondylitis)	One year		*Functional capacity* *^3^* (0-10): MD –0.61 (95% CI –1.38 to 0.16) favoring GPR *Disease activity* *^2^* (0-10): MD –0.06 (95% CI –0.83 to 0.71) favoring GPR			
Silva et al.^30^ (Ankylosing Spondylitis)	16 weeks		*Pain (0-10)* *Cervical pain*: MD 1.3 (95% CI 1.02 to 1.57) favoring the control condition^*^ Dorsal pain: MD 0.1 (95% CI –0.15 to 0.35) favoring the control condition Lumbar pain: MD –0.5 (95% CI –0.77 to –0.22) favoring GPR^*^ Functional capacity: MD –0.60 (95% CI –0.68 to –0.51) favoring GPR^*^ Disease activity: MD –1.4 (95% CI –1.59 to –1.20) favoring GPR^*^		GPR did not reduce cervical and dorsal pain compared to the control intervention. Lumbar pain significantly reduced for the GPR group. Functional capacity and disease activity significantly improved in the GPR group compared to the control intervention.	

MD: mean difference; GPR: Global Posture Reeducation; CI: confidence interval; SS: segmental stretching; TMJ: temporomandibular joint; 1. BASFI: Bath Ankylosing Spondylitis Functional Index; 2. BASDAI: Bath Ankylosing Spondylitis Disease Activity Index; 3. Roland-Morris disability questionnaire; 4. NDI, Neck Disability Index; 5. Lysholm Knee Questionnaire.

* Statistically significant difference between groups.

#### Pregnancy-related low back pain

In the trial by Gil et al.^22^, GPR was more effective than no treatment for pain and disability reduction, and the effect was clinically relevant in both outcomes. There was very low quality evidence (GRADE) from a single study with high risk of bias that GPR significantly reduced pain and improved disability in patients with pregnancy-related low back pain.

#### Chronic low back pain

In the trial by Lawand et al.^35^, GPR was more effective than no intervention for pain and disability reduction at twelve weeks and six months. However, pain reduction was clinically relevant only at twelve weeks. There was low quality evidence (GRADE) from a single study with low risk of bias that GPR reduced pain and disability compared to no treatment for chronic low back pain.

#### Ankylosing Spondylitis

In the trial by Durmus et al.^21^, GPR was more effective than no treatment for pain and disease activity reduction, but only pain reduction was clinically relevant. There was very low quality evidence (GRADE) from a single study with high risk of bias showing that GPR significantly reduced pain and disease activity and did not change functional capacity status in patients with ankylosing spondylitis.

### GPR versus segmental stretching alone

Four studies^4^ ^,^ ^5^ ^,^ ^20^ ^,^ ^32^ comprising 121 patients, of which 105 (86.77%) were followed-up, compared GPR to segmental stretching alone in neck pain^4^ ^,^ ^32^, patellofemoral pain syndrome^20^, and temporomandibular disorders (Table 3)^5^.

#### Neck pain

**Figure 2:**
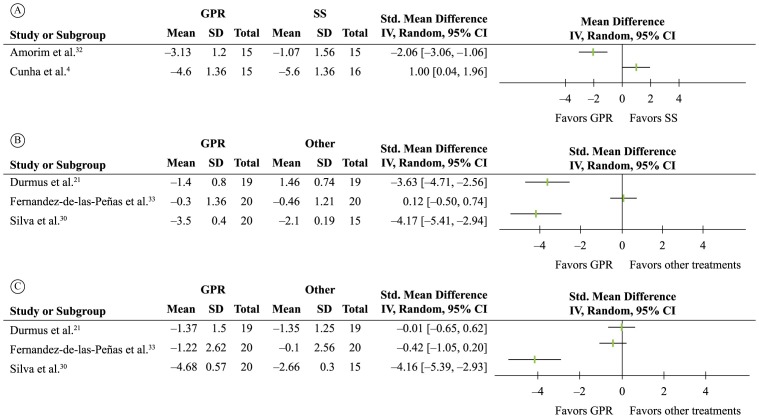
Descriptive forest-plots (pooling was not possible due to very high levels of statistical heterogeneity) for the outcomes (A) pain in trials comparing GPR versus segmental stretching for neck pain; (B) functional capacity in trials comparing GPR versus other treatments for ankylosing spondylitis; (C) disease activity in trials comparing GPR versus other treatments for ankylosing spondylitis.

In the trial by Amorim et al.^32^, GPR was more effective than segmental stretching for pain and disability reduction, whereas in the trial by Cunha et al.^4^, segmental stretching was more effective than GPR at six and twelve weeks for pain reduction. Both trials had high risk of bias as well as imprecise and inconsistent findings, therefore the evidence was rated as “very low quality” (GRADE) (Figure 2).

#### Patellofemoral pain syndrome

In individuals with patellofemoral pain syndrome^20^, GPR was not more effective than segmental stretching for pain and disability reduction. There was very low quality evidence (GRADE) from a single trial with high risk of bias that GPR did not reduce pain or disability compared to segmental stretching in patellofemoral pain syndrome.

#### Temporomandibular disorders

In a trial assessing the effects of GPR versus segmental stretching in patients with temporomandibular disorders^5^, GPR was not more effective than segmental stretching for temporomandibular joint pain. There was very low quality evidence (GRADE) from a single trial with high risk of bias that GPR was not more effective than segmental stretching alone for pain reduction in temporomandibular disorders.

### GPR versus other treatments

Five trials, involving 152 patients, among which 143 were followed up (94.07%) compared GPR to other treatment strategies in chronic low back pain^31^ and ankylosing spondylitis (Table 3)^21^ ^,^ ^30^ ^,^ ^33^ ^,^ ^34^.

### Chronic low back pain

A single trial showed that GPR was not more effective than Isostretching for pain reduction at twelve and twenty months^31^. There was very low quality evidence (GRADE) from a single trial with high risk of bias that GPR was not more effective than Isostretching for pain reduction in chronic low back pain.

### Ankylosing Spondylitis

Four trials^21^ ^,^ ^30^ ^,^ ^33^ ^,^ ^34^ were conducted in participants with ankylosing spondylitis. GPR was not more effective than a comprehensive exercise program in the trial by Durmus et al.^21^ In the trial by Silva et al.^30^, segmental stretching plus breathing exercises were superior to GPR for cervical pain reduction, whereas no effect for dorsal pain and a significant effect for lumbar pain reduction favoring GPR occurred, but none of the differences were clinically relevant. There was very low quality evidence (GRADE) based on two trials with high risk of bias with inconsistent and imprecise results that GPR was more effective than other interventions for pain reduction in patients with ankylosing spondylitis.

Functional capacity and disease activity were assessed in four trials^21^ ^,^ ^30^ ^,^ ^33^ ^,^ ^34^. Overall, there were conflicting and imprecise findings regarding the ability of GPR to improve these outcomes (Figure 2). Based on four trials with high risk of bias, inconsistent and imprecise findings, evidence that GPR did significantly improve functional capacity and did not reduce disease activity compared to other intervention was rated as “very low quality” (GRADE).

## Discussion

This systematic review showed that GPR was more effective than no treatment for pain and disability in pregnancy-related low back pain, chronic low back pain, and ankylosing spondylitis, but not for disease activity in ankylosing spondylitis. Conversely, GPR was not more effective for pain and disability reduction in patellofemoral pain syndrome, temporomandibular disorders, and neck pain. Likewise, GPR was not more effective than other treatments for pain in chronic low back pain and ankylosing spondylitis, as well as for functional capacity and disease activity in ankylosing spondylitis. Some of the presented findings appear to reflect the current knowledge on the therapeutic effect of exercise modalities on musculoskeletal conditions. For instance, Yamato et al.^36^ found that Pilates is probably more effective than minimal intervention and probably not more effective than other exercises for pain and disability reduction in patients with low back pain. Likewise, Dagfinrud et al.^37^ found that either home or supervised exercises were superior to no treatment for physical function improvement. Overall, it was demonstrated that GPR was superior to no treatment and not superior to segmental stretching alone or other treatment regimens in patient-reported outcomes.

GPR compared to no treatment resulted in clinically important differences in some outcomes. Patients with pregnancy-related low back pain undergoing GPR^22^ achieved a between-group reduction of 5.5 points in pain (visual analogue scale) and 9.10 points in disability (Rolland-Morris questionnaire), which is far beyond the minimum clinically important difference values for each outcome measure, set at 1.5 and 5 points, respectively^38^. Patients with chronic low back pain treated with GPR in the trial by Lawand et al.^35^ had a between-group reduction of 3.1 points in pain (visual analogue scale) at three months, which is considered to be a clinically important change. At six months, however, this difference was no longer clinically important^38^. Conversely, the point estimate for disease activity in the Durmus et al.^21^ trial did not reach the minimum clinically important difference (1 point for the Bath Ankylosing Spondylitis Disease Activity Index)^39^, but the confidence interval included some clinically important effects.

GPR compared to segmental stretching alone for neck pain in the Amorim et al.^32^ trial resulted in a between-group reduction of 2.06 points in pain and 7.3 points in disability (Neck Disability Index) favoring GPR, whose minimum clinically important difference is 3.5 points. This finding must be interpreted with caution, as a recent Cochrane Review^40^ found that stretching alone did not result in additional benefit for chronic mechanical neck pain compared to strengthening and endurance training. Therefore, future high-quality trials should compare GPR to effective treatment strategies for neck pain, such as manual therapy or strengthening exercises. In this review, we found no studies demonstrating clinically important benefits of GPR compared to treatment strategies other than segmental stretching alone.

The majority of the included studies was rated as very low quality, which means that there is substantial uncertainty in their results. This might be explained by the high risk of bias inherent to most of the included studies (ten trials failed to address selection, performance, or attrition), the presence of high statistical heterogeneity (which precluded pooling), and the presence of imprecise estimates (little or no overlap between confidence intervals), which downgraded the evidence for within-study limitations, inconsistency, and imprecision, respectively. Conversely, considering that only three studies^5^ ^,^ ^32^ ^,^ ^35^ adequately reported sample size calculation and all studies had small sample sizes, type II error may have emerged in many trials. However, the latter possibility would affect only the direction and the magnitude of the findings, not the quality of the summarized evidence. Furthermore, despite the fact that small sample sizes, lack of outcome assessor blinding, allocation concealment, and intention-to-treat analysis may overestimate the true effect size of an intervention^41^ ^-^ ^44^, all studies had small sample sizes and most studies failed to adequately report these three domains of bias assessment, but only two trials showed large effect sizes favoring GPR^22^ ^,^ ^32^.

This review has limitations. The small number of included studies and participants yielded comparisons derived from single studies, which hampered the generalizability of some findings and the conduction of the pre-planned subgroup analysis. Despite the language restrictions in the selection of studies, this systematic review included a comprehensive search within several databases in order to enhance its sensitivity. Still, some evidence from non-peer reviewed randomized controlled trials may have been missed. Furthermore, it was not possible to undertake meta-analysis due to high levels of statistical heterogeneity and because the majority of the included studies had high risk of bias, and therefore statistical pooling was not recommended for some comparisons^12^.

## Conclusion

GPR was effective for pain and disability reduction in pregnancy-related low back pain, chronic low back pain, and ankylosing spondylitis compared to no treatment, but not superior to segmental stretching and other treatments for pain and disability in neck pain, temporomandibular disorders, and patellofemoral pain syndrome, chronic low back pain, and ankylosing spondylitis. Moreover, GPR was more effective than other treatments for functional capacity improvement but not more effective for disease activity reduction in ankylosing spondylitis. Based on this, characteristics such as patient preferences, care provider’s expertise, preference, and costs should aid the decision to use GPR in these conditions. These results must be interpreted with caution, due to the low to very low quality evidence. It is very likely that future trials should change the estimates of the effect of GPR on patient-reported outcomes in the studied conditions.

## Appendix 1 Search strategy on MEDLINE (via PubMed).

**Table:**

#1 (Intervention)	Global posture reeducation OR global postural reeducation OR postural reeducation OR posture reeducation
	AND
#2 (type of study)	randomized controlled trial [Publication Type] OR controlled clinical trial [Publication Type] OR randomized controlled trials [MeSH Terms] OR random allocation [MeSH Terms] OR double blind method [MeSH Terms] OR single blind method [MeSH Terms] OR clinical trial [Publication Type] OR clinical trials [MeSH Terms] OR (clinical* [Text Word] AND trial* [Text Word]) OR single* [Text Word] OR double* [Text Word] OR treble* [Text Word] OR triple* [Text Word] OR placebos [MeSH Terms] OR placebo* [Text Word] OR random* [Text Word] OR research design [MeSH Terms] OR follow-up studies [MeSH Terms] OR prospective studies [MeSH Terms] OR control* [Text Word] OR prospective* [Text Word] OR volunteer* [Text Word]
